# Farnesoid X receptor knockout protects brain against ischemic injury through reducing neuronal apoptosis in mice

**DOI:** 10.1186/s12974-020-01838-w

**Published:** 2020-05-25

**Authors:** Hui-Min Shan, Minhua Zang, Qi Zhang, Ru-Bing Shi, Xiao-Jing Shi, Muyassar Mamtilahun, Chang Liu, Long-long Luo, Xiaoying Tian, Zhijun Zhang, Guo-Yuan Yang, Yaohui Tang, Jun Pu, Yongting Wang

**Affiliations:** 1 School of Biomedical Engineering and Med-X Research Institute, Shanghai Jiao Tong University, 1954 Hua-Shan Road, Shanghai, 200030 China; 2 Department of Cardiology, Renji Hospital, School of Medicine, Shanghai Jiao Tong University, 160 PuJian Road, Shanghai, 200127 China

**Keywords:** Calcium influx, Farnesoid X receptor, Ischemic stroke, Inflammation, Neuronal apoptosis

## Abstract

**Background:**

Farnesoid X receptor (FXR) is a nuclear receptor that plays a critical role in controlling cell apoptosis in diverse diseases. Previous studies have shown that knocking out *FXR* improved cardiac function by reducing cardiomyocyte apoptosis in myocardial ischemic mice. However, the role of FXR after cerebral ischemia remains unknown. In this study, we explored the effects and mechanisms of *FXR* knockout (KO) on the functional recovery of mice post cerebral ischemia-reperfusion.

**Methods:**

Adult male C57BL/6 wild type and *FXR* KO mice were subjected to 90-min transient middle cerebral artery occlusion (tMCAO). The mice were divided into five groups: sham, wild-type tMCAO, *FXR* KO tMCAO, wild-type tMCAO treated with calcium agonist Bayk8644, and *FXR* KO tMCAO treated with Bayk8644. FXR expression was examined using immunohistochemistry and Western blot. Brain infarct and brain atrophy volume were examined at 3 and 14 days after stroke respectively. Neurobehavioral tests were conducted up to 14 days after stroke. The protein levels of apoptotic factors (Bcl-2, Bax, and Cleaved caspase-3) and mRNA levels of pro-inflammatory factors (TNF-α, IL-6, IL-1β, IL-17, and IL-18) were examined using Western blot and RT-PCR. TUNEL staining and calcium imaging were obtained using confocal and two-photon microscopy.

**Results:**

The expression of FXR was upregulated after ischemic stroke, which is located in the nucleus of the neurons. *FXR* KO was found to reduce infarct volume and promote neurobehavioral recovery following tMCAO compared to the vehicle. The expression of apoptotic and pro-inflammatory factors decreased in *FXR* KO mice compared to the control. The number of NeuN^+^/TUNEL^+^ cells declined in the peri-infarct area of *FXR* KO mice compared to the vehicle. We further demonstrated that inhibition of FXR reduced calcium overload and addition of ionomycin could reverse this neuroprotective effect in vitro. What is more, in vivo results showed that enhancement of intracellular calcium concentrations could aggravate ischemic injury and reverse the neuroprotective effect of *FXR* KO in mice.

**Conclusions:**

*FXR* KO can promote neurobehavioral recovery and attenuate ischemic brain injury, inflammatory release, and neuronal apoptosis via reducing calcium influx, suggesting its role as a therapeutic target for stroke treatments.

## Introduction

With the recent improvement of acute ischemic stroke management with tissue plasminogen activator (tPA) and intravascular thrombectomy, a revisit of neuroprotection treatment in combination with recanalization is likely to provide insights into developing treatment strategies that will provide additional benefits to patients. During an ischemic stroke attack, the inhibition of oxygen and glucose supply leads to the release of glutamate into the extracellular space, producing an influx of calcium into the cell which can activate mitochondrial permeability transition and trigger cell death [1]. Stroke lesions are characterized by a core of necrotic cell death formed rapidly post injury and may represent tissues that are irreversibly lost. However, apoptosis contributes to a significant proportion of neural death in the penumbra where the area is at a high risk of loss but potentially salvageable [2, 3]. Therefore, in the pathological process of ischemic stroke, reducing neuronal apoptosis in the ischemic penumbra holds great potential for stroke therapy [4].

Farnesoid X receptor (FXR) is a nuclear receptor subfamily 1 group H member 4 (NR1H4) which has crucial regulatory functions in multiple biological processes including cholestasis and atherosclerosis [5]. Knockout of *FXR* gene has protective effects in several pathological disorders, including diet-induced obesity and myocardial infarction [6, 7]. GW4064, an agonist of FXR, has been reported to activate permeability transition pores and promote cytochrome c release together with stimulation of caspase-9 and caspase-3 in myocardial ischemia/reperfusion injury, resulting in worsened outcomes [8, 9].

Previous studies focused on the therapeutic effect of *FXR* knockout on different organs such as the liver, kidneys, and intestines where FXR is highly expressed, but little is known regarding its function in the brain [10, 11]. Recently, one study found that *FXR* knockout mice showed increased motor activity but less depressive-like and anxiety-related behaviors. The deletion of *FXR* increased GABA transporter 1 (GAT1) expression in the cerebral cortex but reduced the amount of the glutamic acid decarboxylase 65 (GAD65) in the hippocampus. In addition, as the most specific endogenous agonists, bile acids (BAs) can bind to FXR and activate it. One of the primary functions of FXR activation is the suppression of cholesterol cytochrome P450 7A1 (CYP7A1), a rate-limiting enzyme in the process of BAs synthesis from cholesterol, through a negative feedback mechanism. BAs have neuroprotective roles in neurodegenerative diseases, through reducing apoptosis and restoring mitochondrial function [12, 13]. Z-guggulsterone (Z-GS), an inhibitor of FXR, can improve the scopolamine-induced memory impairment through enhancement of the signalling of brain-derived neurotrophic factor (BDNF) [14]. These series of agonists and antagonists provide valuable tools in studying the effect of *FXR* KO on inflammation, apoptosis, and neurobehavioral outcomes after ischemic stroke.

In this study, we seek to investigate the effect of *FXR* KO in ischemic brain injury using a knockout mouse model of ischemic stroke. We also investigated the potential mechanisms of FXR through the use of FXR antagonists in cultured wild-type primary neurons.

## Methods

### Animals

**Fig. 1:**
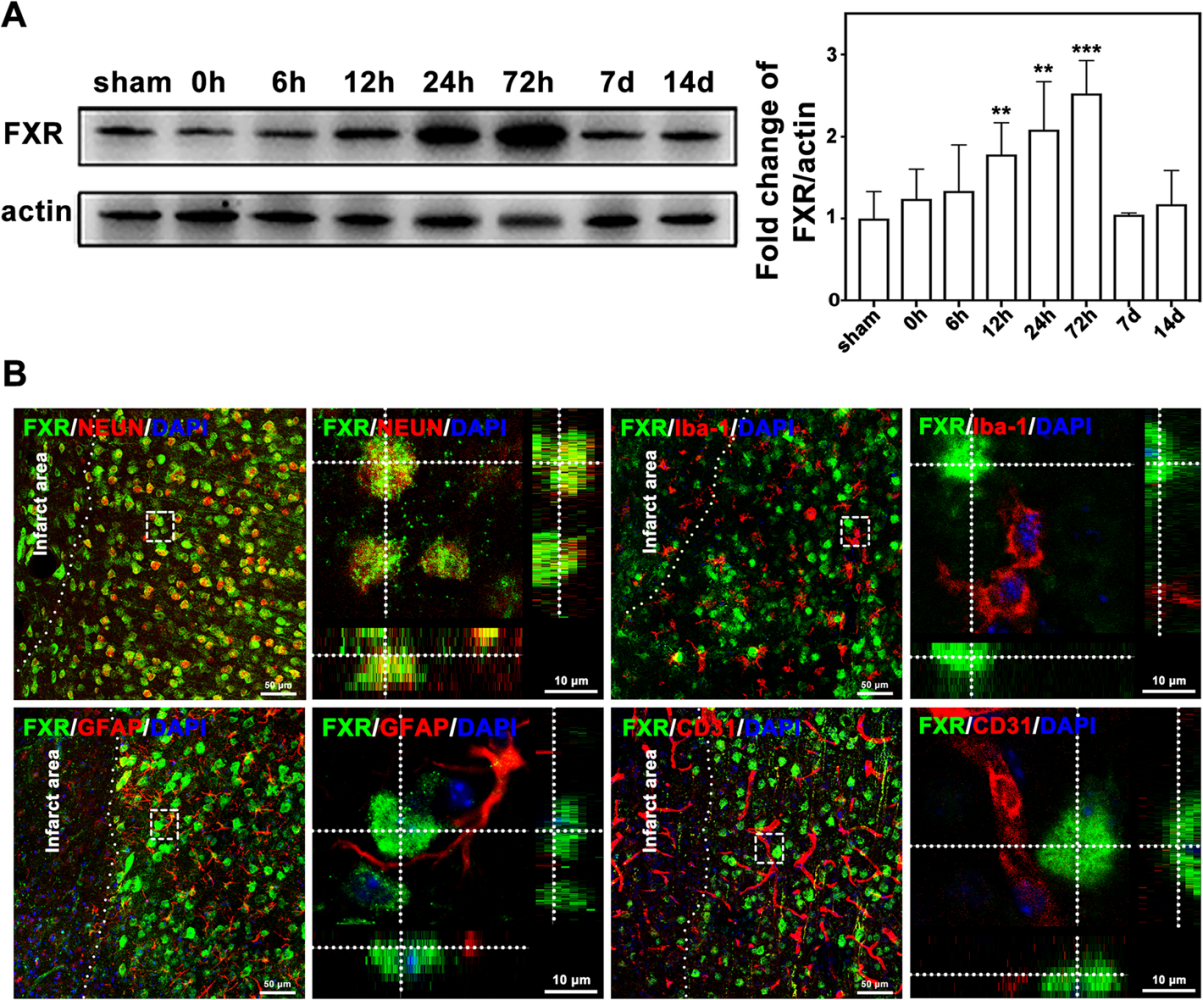
FXR was upregulated in neurons after cerebral ischemia. **a** Western blot of FXR expression in the mouse brain at different time points after tMCAO. Data are presented as mean ± SD, *n* = 6–8/group, **p < 0.01, ***p < 0.001. **b** Confocal imaging showed the co-localization of FXR (green) with neurons (NeuN, red), microglia (Iba-1, red), astrocytes (GFAP, red), and endothelial cells (CD31, red) in the peri-infarct area at 3 days after stroke, Bar = 50 μm and 10 μm, respectively

Animal protocols were approved by the Institutional Animal Care and Use Committee (IACUC) of Med-X research institute, Shanghai Jiao Tong University, Shanghai, China and animal work in this study was performed in accordance with ARRIVE guidelines. A total of 175 (20–25 g, 8–10 weeks old) male C57BL/6 mice purchased from JSJ laboratory (JSJ laboratory, Shanghai, China) and 38 *FXR* knockout male mice (20–25 g, 8–10 weeks old) from C57/BL6 background purchased from Jackson laboratory (Jackson Lab, Sacramento, CA, USA) were housed in standard 12 h light/dark cycle in transparent cages (five or less per cage) with free access to food and water and maintained in an ambient temperature of 21∼25 °C and a humidity of 20∼50%. Wild-type C57BL/6 mice were randomly assigned to sham (*n* = 25), vehicle (*n* = 135), and Bayk8644 treatment (*n* = 15) groups, while *FXR* knockout mice were randomly divided into *FXR* knockout (*n* = 23) and *FXR* knockout plus Bayk8644 treatment (*n* = 15) groups. The experimental designs are summarized in Figs. 1a and 6a.

### Transient middle cerebral artery occlusion surgery

The surgical procedure for transient middle cerebral artery occlusion (tMCAO) has been previously described [15]. Briefly, animals were anesthetized with 1.5% isoflurane in a 30% O^2^/70% NO mixture under spontaneous breathing conditions on a heating pad (RWD Life Science, Shenzhen, China). A 6-0 suture (Covidien, Mansfield, MA, USA) coated with silicon was inserted through the external carotid artery (ECA) with an advancement of 0.9–0.95 mm from the internal carotid artery (ICA) until reaching the intersection of the middle cerebral artery (MCA). After 90 min of ischemia, the suture was withdrawn to allow reperfusion. A laser Doppler Monitor (Moor Lab, Moor Instruments, Devon, UK) was used to monitor the blood flow in the MCA territory before surgery, immediately after occlusion, and upon reperfusion. Successful occlusion of the MCA was defined as a decline in the regional blood flow of the ipsilateral hemisphere to 20% of the baseline. A recovery of cerebral blood flow to more than 70% of the baseline was regarded as a successful reperfusion. The mortality rate of mice that underwent tMCAO was 7.6%. Animals were randomly assigned into different groups. Both the surgeon and the behavioral analyst were blinded to treatment assignments.

### Drug administration

In order to investigate the biological function of FXR in vivo, an L-type calcium channel agonist, Bayk8644 (2 mg/kg, Meilunbio, Dalian, China) was given intraperitoneally at 10 min after reperfusion. Bayk8644 stock solution (1 mmol/L in dimethyl sulfoxide, DMSO) was diluted in 1× phosphate-buffered saline (PBS) with a final DMSO concentration of 4.6%. Similarly, PBS with 4.6% DMSO was given intraperitoneally in sham, wild-type vehicle, and *FXR* knockout vehicle groups as control. To further explore dose response in primary neurons, a dose range of ionomycin (50 nmol/L, Beyotime Biotechnology, Shanghai, China), GW4064 (0, 1, 1.5, 3, and 6 μmol/L, Selleck, Houston, USA), or Z-guggulsterone (Z-GS, 0, 2.5, 5, and 10 μmol/L, Thermo Scientific, Waltham, MA) was given for 18 h before oxygen and glucose deprivation (OGD).

### Neurobehavioral assessment

Neurological behavioral tests were carried out before tMCAO and at 1, 3, 7, and 14 days after tMCAO by another investigator blind to the experimental groups using the modified neurological severity score (mNSS), rotarod test, and hanging wire test.

mNSS of the animals were graded on a scale of 0 to 14, which is a composite of motor, sensory, and balance tests [16].

The rotarod test is an index of sensorimotor coordination and motor learning in rodent models of central nervous system (CNS) disorders [17]. Three days of training prior to surgery were carried out to ensure that all mice have learned the task to the same degree. During training, mice learnt to balance on a stationary rod, then on a rod constantly rotating at 20 rpm. In the rotarod test performed after surgery, the mice were placed on a rod which accelerated to 40 rpm in 2 min and then constantly rotated at 40 rpm for another 3 min. The latency to fall was recorded. A trial was deemed complete when the animal falls or the time period ends. Each animal was given four trials, and the time that the animals spent on the rungs or gripped the device and spun around for two consecutive revolutions was recorded. Data were analyzed as the average duration of four trials on the rotarod.

Hanging wire test was performed in order to evaluate a motor neuromuscular impairment and motor coordination [18]. In this method, mice were subjected to a 180-s hanging test, during which a “falling” score or “reaching” score was diminished or increased by 1, respectively. Reaching and falling scores were recorded in 180 s as well as the elapsed times between falls. The impulse, which is the weight (in grams) times time between falls (in seconds), was also the final outcome measure.

### Infarct volume and atrophy measurement

Mouse brains were transcardially perfused with 0.9% saline and 4% paraformaldehyde (PFA, Sinopharm Chemical Reagent, Shanghai, China). After perfusion, brains were removed and then placed in − 80 °C isopentane for 20 s and then stored at − 80 °C. A series of 20-μm-thick coronal brain sections were collected for a total thickness of nearly 5.2 mm. No.1, 11, 21,…, 251 brain slices were collected to determine infarct volume using a 0.1% Cresyl violet solution (Meilunbio), and the ratio of staining in the ipsilateral and contralateral hemispheres was calculated using ImageJ (National Institutes of Health, Bethesda, MD). The calculation of brain infarction volume was performed by using the following formula: *V* =∑*h*/3[∆S_n_+(∆S_n_*∆S_n+1_)^1/2^+∆S_n+1_], where *V* represents volume, *h* represents the distance between the two adjacent brain sections (*h* = 0.02 mm), contralateral area minus the normal area of the ipsilateral hemisphere was recorded as the infarct area ΔS, and the infarction area of two adjacent pieces is denoted as ∆S_n_ and ∆S_n+1_. In addition, the measurement of brain atrophy volume was calculated by using contralateral area minus the ipsilateral area by the same method above.

### Immunostaining and quantification

After treatment with 0.3% Triton X-100 for 10 min, brain sections or cells were blocked for 60 min in 1% bovine serum albumin (BSA) at room temperature. The samples were then incubated with primary antibodies against FXR (1:100; Santa Cruz, CA, USA), glial fibrillary acidic protein (GFAP) (1:200, Billerica, MA, USA), NeuN (1:200, Millipore, Billerica, MA, USA), MAP2 (1:200, Millipore), cluster of differentiation 31 (CD31) (1:200, R&D Systems, Minneapolis, MN, USA), or ionized calcium binding adaptor molecule-1 (Iba-1) (1:200, WAKO, Osaka, Japan) overnight at 4 °C. After being washed three times with PBS for 10 min, sections or cells were incubated with secondary antibody for 1 h at 37 °C and then 4′,6-diamidino-2-phenylindole dihydrochloride (DAPI) (1:1000, Beyotime). Apoptosis of neurons was evaluated by terminal-deoxynucleotidyl transferase mediated nick end labelling (TUNEL) and NeuN double immunostaining according to the manufacturer’s protocol (Meilunbio). Four brain sections that were 200 μm apart from each brain sample were chosen for TUNEL staining, and then stained with anti-NeuN antibody. The apoptotic cells were calculated from five areas in the striatum and cortex of the peri-infarct area from the aforementioned four brain sections by two observers blinded to the experimental group using a fluorescent microscope (Leica, Solms, Germany). For in vitro apoptosis analysis, the percentage of TUNEL-positive cells was calculated by dividing the number of DAPI/TUNEL double-positive cells over the total number of DAPI-positive cells in five random regions of each cell slide in each group.

### Western blotting analysis

At 0 h, 6 h, 12 h, 24 h, 72 h, 7 days, and 14 days after stroke, proteins were extracted from the ipsilateral hemisphere of the striatum of the mice and placed in RIPA Lysate (Millipore). The Western blot technique was performed as previously described [19, 20]. Briefly, protein content was quantified using bicinchoninic acid (BCA) protein assay (Meilunbio). Equal amount of protein samples were subjected to sodium dodecyl sulfate-polyacrylamide gel electrophoresis (SDS-PAGE) and transferred to PVDF membrane using a Trans-blot apparatus (Bio-Rad, Hercules, CA). The membranes were blocked with 5% non-fat dry milk in tris-buffered saline tween (TBST, Meilunbio) for 1 h at room temperature before being incubated with the primary antibodies overnight at 4 °C. The following primary antibodies were used: FXR (1:750, Santa Cruz), B cell lymphoma-2 (BCL-2) (1:1000, Cell Signaling Technology, Beverly, MA, USA), BCL-2 associated X protein (Bax) (1:1000, Abcam, Cambridge, MA, USA), Cleaved Caspase-3 (1:1000, Cell Signaling technology), and β-actin (1:1000, Santa Cruz) which was employed as the internal control. The corresponding horseradish peroxidase-conjugated goat anti-mouse and goat anti-rabbit (Santa Cruz Biotechnology) were used respectively for chemiluminescence-based Western blot analyses, and the bound secondary antibody signals were as detected using super sensitive chemiluminescence reagent (Meilunbio). For quantification of data from Western blot, band intensities of four to five samples were analyzed. After calculating the intensity value of the band relative to the expression of the internal reference β-actin, the levels were normalized to those of the control group and statistical analysis was performed with ImageJ.

### Reverse transcription-polymerase chain reaction analysis

The total RNA was extracted from the brain tissues in the peri-infarct area of striatum at 72 h following tMCAO using TRIzol (Invitrogen, Carlsbad, CA), and cDNA synthesis was performed using a commercial kit (Hifair^TM^ II 1st Strand cDNA Synthesis SuperMix for qPCR, QIAGEN, Hilden, Germany) according to the manufacturer’s instructions [21, 22]. Quantitative PCR was conducted using a commercial mix (SYBR Green Master Mix, QIAGEN) and tested by a fast reverse transcription-polymerase chain reaction (RT-PCR) system (7900 HT, ABI, Foster City, CA). The qPCR amplification reaction was carried out in a final volume of 20 μl, containing of 10 μl Master Mix, 0.5 μl of each primer (10 μM), 8 μl water, and 50 ng of cDNA. The amplification was as follows: denaturation at 95 °C for 10 min, 40 PCR cycles of 95 °C for 5 s, 60 °C for 30 s, followed by 1 cycle at 95 °C for 15 s, 60 °C for 1 min, and 95 °C for 15 s with continuous fluorescence measurement. Quantification was performed by using comparative CT method (2^−ΔΔCT^) and all samples were analyzed in triplicate. Sequence-specific primers for *TNF-α*, *interleukin-6* (*IL-6*), *interleukin-1β* (*IL-1β*), *interleukin-17* (*IL-17*), interleukin-18 (*IL-18*), and *GAPDH* are shown in Table 1. The relative expression was normalized to that of the sham group.

**Table 1:** Sequence-specific primers for TNF-α, IL-6, IL-1β, IL-17, IL-18, and GAPDH were showed as follows

Gene	Forward primer (5′–3′)	Reverse primer (5′–3′)
*TNF-α*	TAGCCAGGAGGGAGAACAGA	CCAGTGAGTGAAAGGGACAGA
*IL-6*	ACCAAGACCATCCAATTCATC	CTGACCACAGTGAGGAATGTC
*IL-1β*	TACATCAGCACCTCACAAGC	AGAAACAGTCCAGCCCATACT
*IL-17*	GTTCGTGCTATTGATTTTCAGC	GGACCCCTTTACACCTTCTTT
*IL-18*	AGGACACTTTCTTGCTTGCCA	CACAAACCCTCCCCACCTAAC
*GAPDH*	AAATGGTGAAGGTCGGTGTG	AGGTCAATGAAGGGGTCGTT

### Cell culture and viability assessment

Previous studies have suggested that rats are physiologically more similar to humans [23]. Also, in order to obtain a large number of neurons in the same batch of experiments, primary neurons were prepared from cerebral cortices of P0 rats [24]. Dissociated cortical cells were spread on 6-well plates, 24-well plates, or 96-well plates coated with poly-d-lysine (Sigma-Aldrich, San Louis, MO, USA) and cultured in Dulbecco’s modified eagle medium (DMEM, HyClone, Logan, UT) with 10% fetal bovine serum (FBS, Gibco, Carlsbad, NM, USA) and 0.5% penicillin and streptomycin antibiotics (HyClone) at a density of 7 × 10^5^ cells/well on 6-well plates, 2 × 10^5^ cells/well on 96-well plates, or 5 × 10^4^ cells/well on 96-well plates. At 4 h after seeding, the medium was changed to Neurobasal-A medium (Gibco) supplemented with B-27 (Gibco). Cells were cultured in a humidified incubator at 37 °C with 5% CO_2_. Cultures were used for experiments at 7 to 10 days after seeding. OGD experiments were performed using a specialized sealed chamber containing an anaerobic gas mixture (95% N_2_ and 5% CO_2_). Culture medium was also replaced with deoxygenated glucose-free Neurobasal-A medium (Gibco). After a 30-min challenge, cultures were removed from the chamber, and the medium in the cultures was replaced with maintenance medium. Cells were then allowed to recover for 12 h in a regular incubator. Cell survival assays were performed by using Cell Counting Kit-8 solution (Dojindo, Kumamoto, Japan). The cell-counting kit-8 (CCK8) solution was added to each well and was incubated at 37 °C for 2 h followed by data collection. Absorbance was measured at 450 nm using a microplate reader (BioTek).

### Two-photon calcium imaging

Cells were planted on 35 mm dishes (World Precision Instruments, Sarasota, FL, USA). After OGD and reoxygenation (OGD/R) for 12 h, cells were washed with calcium-free PBS and loaded with 0.25 μM Fluo-4 AM (Sigma) in Neurobasal-A medium plus B27 (calcium-free) for 30 min at 37 °C. The cells were then washed with PBS twice and incubated in Neurobasal-A medium plus B27 for an additional 30 min. Images were captured by an inverted two-photon microscope (Nikon, Kyoto, Japan) to detect the fluorescence intensity of cells, which represented the calcium concentration in the cytoplasm, and subsequently analyzed using ImageJ software (National Institutes of Health, Bethesda, Maryland, USA).

### Statistical analysis

The parametric data were analyzed using Prism GraphPad 8. One-way ANOVA followed by Tukey post hoc tests was used for statistical comparisons among multiple groups. Comparisons between two groups were made by Student’s *t* test. All data were expressed as mean ± standard deviation (SD); a *p* value less than 0.05 was considered statistically significant [25].

## Results

### The expression of FXR was upregulated in neurons after cerebral ischemia

To investigate the change of FXR expression post stroke, Western blot and immunohistochemical analyses were performed at different time points in ischemic brain. Western blot confirmed that the expression of FXR in the ischemic penumbra started to increase at 12 h after stroke (*p* < 0.01), reaching its peak at 72 h (*p* < 0.001), and returned to similar level of the sham group at 7 days and 14 days after stroke (Fig. 1a). To determine the cellular source of FXR, FXR/NeuN, FXR/Iba-1, FXR/GFAP, and FXR/CD31 double staining were conducted. The results revealed that FXR was expressed only in the nucleus of neurons rather than in microglia, astrocytes, or endothelial cells (Fig. 1b).

### *FXR* knockout reduced brain infarct volume and promoted neurobehavioral recovery after stroke

**Fig. 2:**
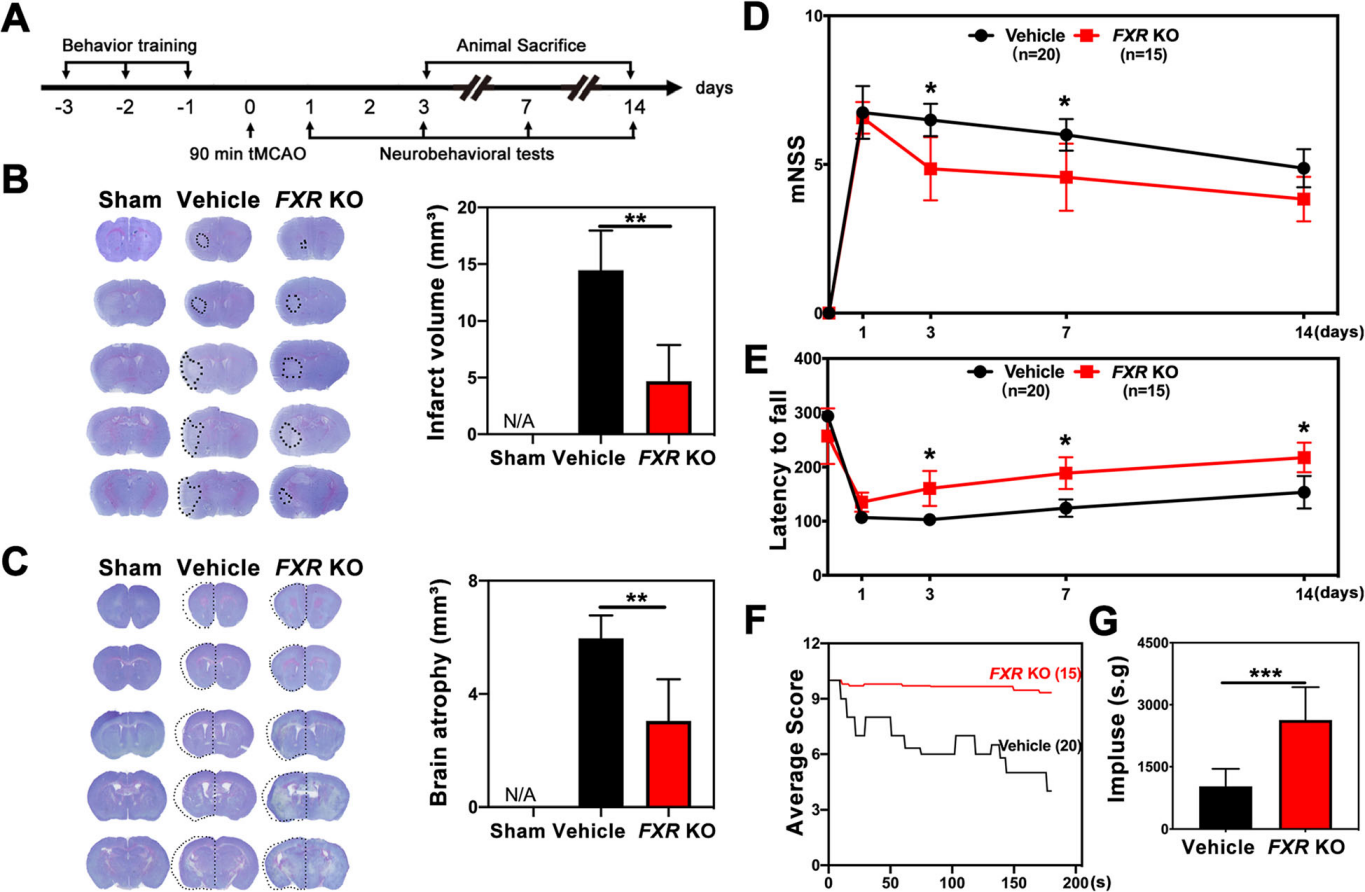
Knockout of *FXR* reduced brain infarct volume and promoted neurobehavioral recovery in mice. **a** Experimental scheme. **b** Representative cresyl violet-stained brain sections at 3 days of sham mice (Sham group), stroke mice treated with vehicle (vehicle group), and *FXR* knockout stroke mice (*FXR* KO group). *n* = 4–7/group. The brain infarct area was circled by the dashed line, and bar graph showed the quantitative comparison of the infarct volume. Data are presented as mean ± SD. **p < 0.01. **c** Brain atrophy at 14 days in sham group, vehicle group, and *FXR* KO group were also detected by Cresyl violet, the dashed line represented brain atrophy. Bar graph showed the brain atrophy volume. Data are presented as mean ± SD. **p < 0.01. Neurobehavioral recovery was assessed by three separate neurobehavioral tests including the modified neurological severity score (mNSS) (D), rotarod performance tests (**e**), and hanging wire tests (**f**, **g**), *n* = 15–20 per group. Data are presented as mean ± SD. *p < 0.05, ***p < 0.001

Our experiment was conducted following the experimental design illustrated in Fig. 2a. We found that 3 days post stroke, knockout of *FXR* (*FXR* KO mice) (4.7 ± 3.2 mm^3^) was associated with reduced brain infarct volume compared to the wild type vehicle group (14.5 ± 3.5 mm^3^) (Fig. 2b). Also, knockout of *FXR* (3.0 ± 1.5 mm^3^) reduced brain atrophy at 14 days after stroke compared to the wild-type mice (6.0 ± 0.8 mm^3^) (Fig. 2c). We further performed neurobehavioral tests to explore the effect of FXR on neurobehavioral recovery following stroke. We found that knockout of *FXR* attenuated neurobehavioral deficits at 3, 7, and 14 days after tMCAO compared to the wild-type vehicle group, as shown by the rotarod test (Fig. 2e). In addition, *FXR* KO mice showed better performance in mNSS (Fig. 2d) at 3 and 7 days after stroke (*p* < 0.05) and in hanging wire test (Fig. 2f–g) at 3 days after stroke (*p* < 0.001)

### Knockout of *FXR* reduced inflammation after ischemic injury

**Fig. 3:**
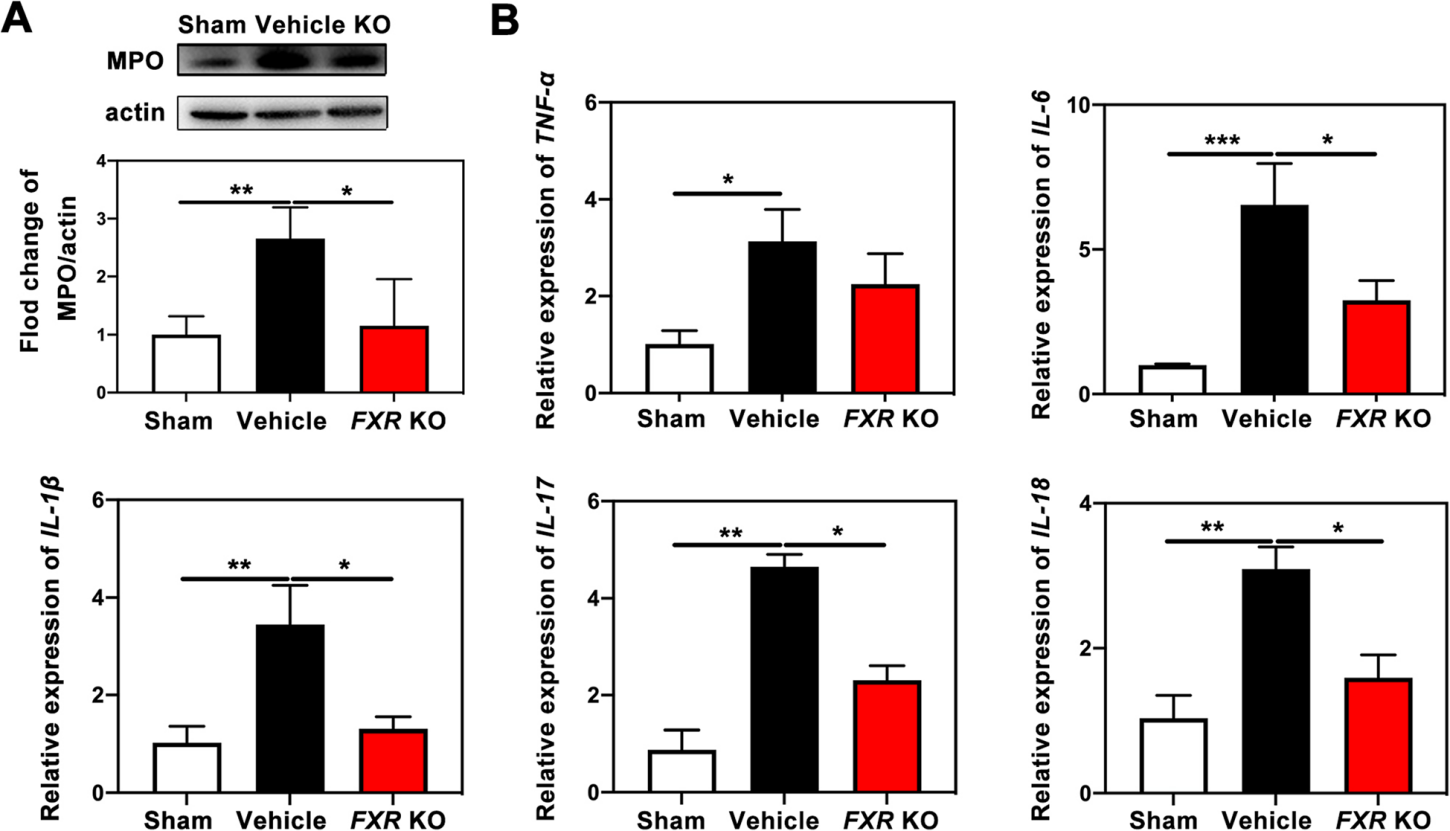
*FXR* knockout reduced inflammation after ischemic injury. **a** Western blotting analysis of MPO in the striatum of ipsilateral hemisphere of sham mice (sham group), stroke mice treated with vehicle (vehicle group) and *FXR* knockout stroke mice (*FXR* KO group) at 3 days after stroke. Data are presented as mean ± SD, *n* = 4–5/group, *p < 0.05, **p < 0.01. **b** The mRNA level of *TNF-α*, *IL-6*, *IL-1β*, *IL-17*, and *IL-18* in the striatum of sham group, vehicle group, and *FXR* KO group at 3 days after stroke. Data are presented as mean ± SD, *n* = 4–5/group, *p < 0.05, **p < 0.01, ***p < 0.001

Myeloperoxidase (MPO), a key inflammatory enzyme secreted by activated neutrophils and macrophages/microglia, can generate highly reactive oxygen species to cause additional damage in cerebral ischemia [26]. In our study, we found the expression of MPO increased significantly post stroke as compared to the sham group (*p* < 0.01), while knockout of *FXR* reduced the level of MPO in the peri-infarct area of brain striatum (*p* < 0.05) in comparison with the vehicle group (Fig. 3a). To determine whether *FXR* knockout can downregulate specific inflammatory cytokines, the levels of pro-inflammatory cytokines including *TNF-α*, *IL-6*, *IL-1β*, *IL-17*, and *IL-18* were examined by RT-PCR. The results demonstrated that proinflammatory cytokines *TNF-α* (*p* < 0.05), *IL-6* (*p* < 0.001), *IL-1β* (*p* < 0.01), *IL-17* (*p* < 0.01), and *IL-18* (*p* < 0.01) were increased in wild-type mice subjected to stroke (Fig. 3b). Compared with the vehicle group, knockout of *FXR* lowered the levels of *IL-6* (*p* < 0.05), *IL-1β* (*p* < 0.05), *IL-17* (*p* < 0.05), and *IL-18* (*p* < 0.05).

### *FXR* knockout prevented apoptosis after cerebral-ischemia

**Fig. 4:**
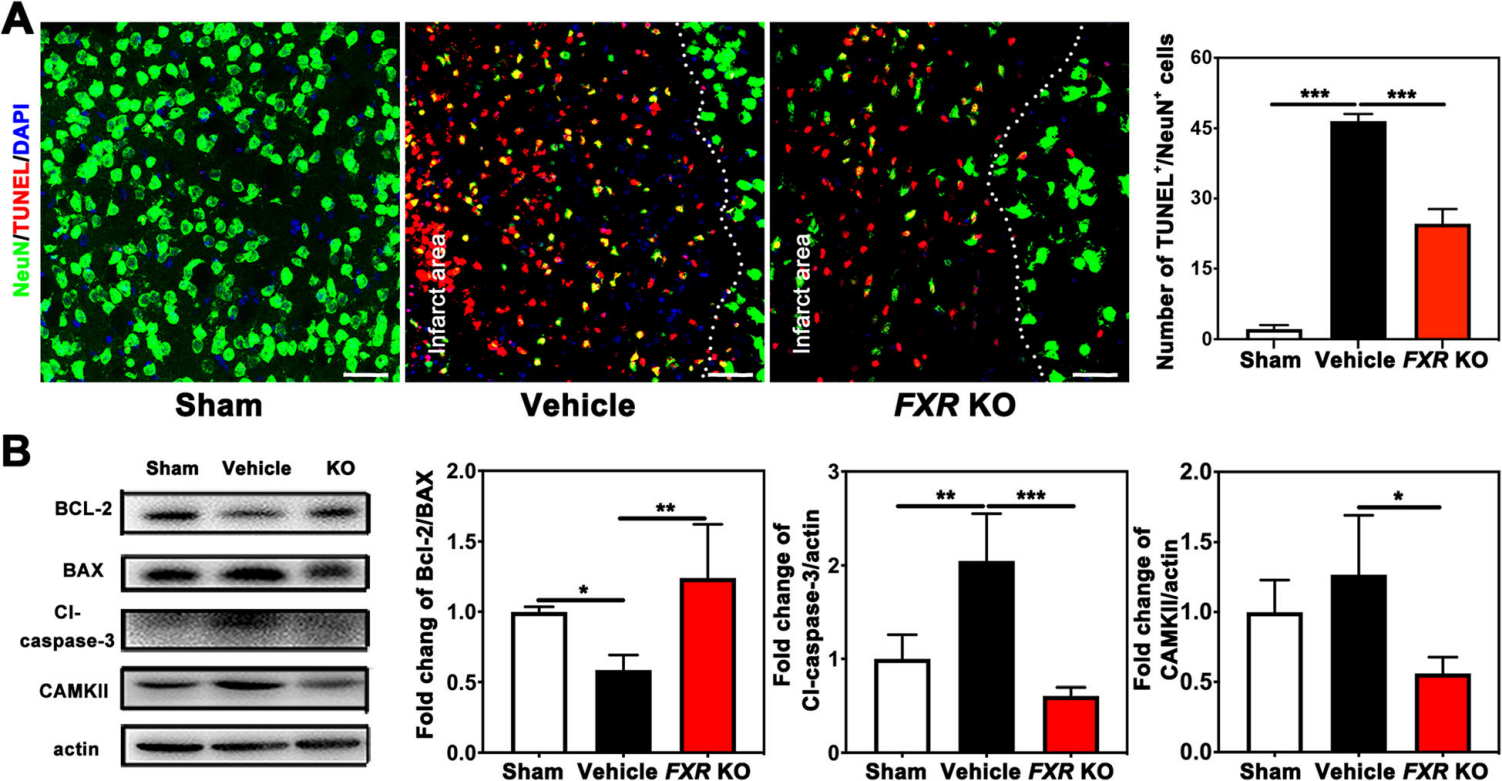
*FXR* knockout prevented apoptosis after cerebral-ischemia. **a** Representative confocal images of NeuN^+^/TUNEL^+^ cells in the brain of peri-infarct area of sham mice (sham group), stroke mice treated with vehicle (vehicle group), and *FXR* knockout stroke mice (*FXR* KO group), Bar = 50 μm. *n* = 4–6 per group. ***p < 0.001. **b** Western blotting analysis of BCL-2, BAX, cleaved cascape-3, and CAMKII in the striatum of ipsilateral hemisphere of sham group, vehicle group, and *FXR* KO group at 3 days after stroke. *n* = 4–5 per group. *p < 0.05, **p < 0.01, ***p < 0.001

To test whether knockout of *FXR* could reduce neuronal apoptosis after cerebral ischemia, brain sections were double stained with TUNEL and NeuN. We found that after 3 days of tMCAO, knockout of *FXR* reduced the number of apoptotic neurons in the penumbra of ischemic mice (24.6 ± 3.1 of *FXR* KO group vs. 46.5 ± 1.5 of vehicle group, *p* < 0.001) (Fig. 4a). In addition, Western blot results showed that knockout of *FXR* increased Bcl-2/Bax ratio (1.2 ± 0.4 of *FXR* KO group vs. 0.5 ± 0.1 of vehicle group, *p* < 0.01) and reduced cleaved caspase-3 expression (0.6 ± 0.1 of *FXR* KO group vs. 2.0 ± 0.5 of vehicle group, *p* < 0.001) when compared to the vehicle group (Fig. 4b). The Ca^2+^/calmodulin (CaM)-dependent protein kinase II (CaMKII) and other members of the CaM kinase family have been implicated in the regulation of both neuronal apoptosis and survival, since the activation of Ca^2+^/calmodulin (CaM)-dependent protein kinase II (CAMKII) can lead to deleterious Ca^2+^ overload and result in cell death [27]. Our results showed that knockout of *FXR* reduced the expression of CAMKII compared to the vehicle group (0.5 ± 0.2 of *FXR* KO group vs. 1.3 ± 0.4 of vehicle group, *p* < 0.05).

### Inhibition of FXR by Z-GS attenuated neuronal apoptosis through reducing OGD-induced calcium influx

**Fig. 5:**
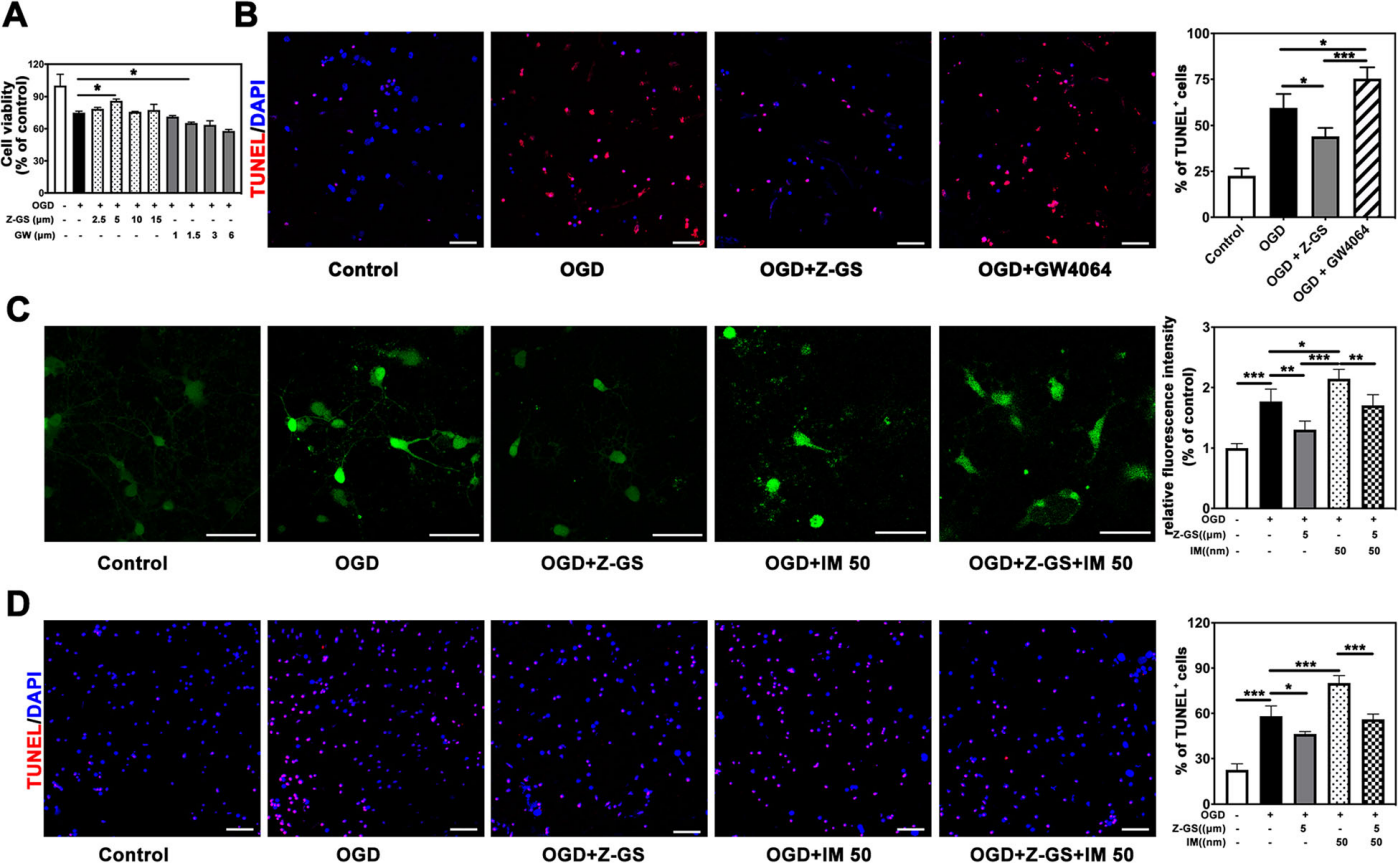
Inhibition of FXR by Z-GS attenuated neuronal apoptosis through reducing OGD-induced calcium influx. **a** Cell viability of primary neurons that subjected to OGD and treated with different concentration of Z-GS and GW4064. *n* = 4–5 per group. *p < 0.05. **b** Double staining of TUNEL (red) and DAPI (blue) of neurons that subjected to OGD and treated with Z-GS (OGD + Z-GS) or GW4064 (OGD + GW4064), Bar = 50 μm. Data are presented as mean ± SD. *n* = 5 per group. *p < 0.05, ***p < 0.001. **c** Calcium images of neurons that subjected to OGD after treatment with Z-GS or IM (50 nM), Bar = 50 μm, *n* = 4–5 per group. *p < 0.05, **p < 0.01, ***p < 0.001. **d** Staining of TUNEL (red) of neurons that subjected to OGD after treatment with Z-GS and ionomycin (IM), Bar = 50 μm. Data are presented as mean ± SD. *n* = 4–5 per group. *p < 0.05, ***p < 0.001

To investigate the effect of FXR on neuronal apoptosis in vitro, we treated primary neurons with Z-GS, an inhibitor of FXR, for 18 h and then performed OGD for 30 min. After reoxygenation for 12 h, a CCK8 assay showed that Z-GS treatment improved the survival of neurons beginning at the concentration of 5 μM (*p* < 0.05), while GW4064 treatment increased neuronal apoptosis which started at the dose of 1.5 μM (*p* < 0.05) (Fig. 5a). Furthermore, the number of TUNEL-positive cells was lessened in Z-GS treated group (5 μM) (44.1 ± 4.6 of Z-GS group vs. 59.7 ± 7.5 of OGD group, *p* < 0.05), and the number of apoptotic cells were increased in GW4064 treatment group (1.5 μM) (75.4 ± 6.1 of GW4064 group vs. 59.7 ± 7.5 of OGD group, *p* < 0.05) (Fig. 5b). Therefore, inhibition of FXR by Z-GS reduced neuronal apoptosis caused by OGD, while the enhanced expression of FXR increased OGD-induced neuronal apoptosis.

To investigate whether inhibition of FXR protected neurons from OGD-induced injury by reducing calcium influx, calcium imaging and TUNEL staining were performed. We found that Z-GS treatment significantly inhibited the rise of cytoplasmic Ca^2+^ induced by OGD (1.3 ± 0.1 of OGD + Z-GS group vs. 1.8 ± 0.2 of OGD group, *p* < 0.01), while IM treatment increased cytoplasmic Ca^2+^ production (1.7 ± 0.2 of OGD + IM group vs. 1.3 ± 0.1 of OGD group, *p* < 0.05). Furthermore, addition of Z-GS in OGD + IM group showed less cytoplasmic Ca^2+^ than OGD + IM group (*p* < 0.01) (Fig. 5c). TUNEL staining demonstrated that Z-GS treatment reduced neuronal apoptosis (46.3 ± 1.7 of OGD + Z-GS group vs. 58.2 ± 6.8 of OGD group, *p* < 0.05) and addition of IM significantly increased apoptotic neurons compared to OGD group (80.1 ± 4.9 of OGD + IM group vs. 58.2 ± 6.8 of OGD group, *p* < 0.001) (Fig. 5d), suggesting that inhibition of FXR can reduce neuronal apoptosis through reducing calcium influx after OGD/R.

### Bayk8644 treatment reversed the protective effects of *FXR* knockout after stroke in mice

**Fig. 6:**
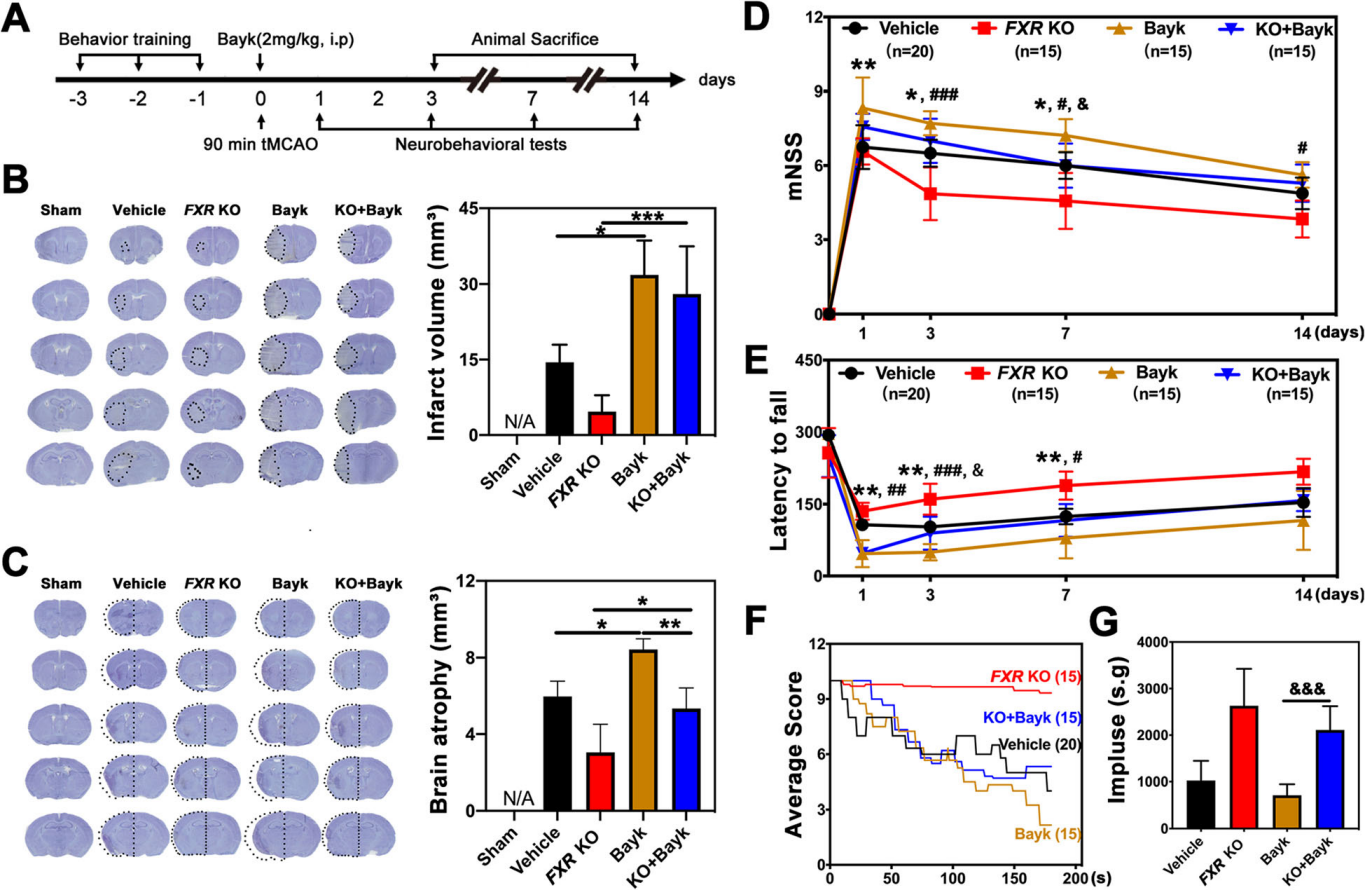
Bayk8644 treatment reversed the protective effects of *FXR* knockout after stroke in mice. **a** Experimental scheme. **b** Representative cresyl violet-stained brain sections at 3 days of sham mice (sham group), stroke mice treated with vehicle (vehicle group), *FXR* knockout stroke mice (*FXR* KO group), wild-type stroke mice treated with Bayk8644 (Bayk group), and *FXR* knockout stroke mice treated with Bayk8644 group (KO + Bayk group). *n* = 4–7/group. The brain infarction was circled by the dashed line, and bar graph showed the quantitative analysis of the infarct volume. Data are presented as mean ± SD. *p < 0.05, ***p < 0.001. **c** Brain atrophy at 14 days in sham group, vehicle group, *FXR* KO group, Bayk group, and KO + Bayk group were also detected by Cresyl violet; the dashed line represented brain atrophy. Bar graph showed the brain atrophy volume, Data are presented as mean ± SD. *p < 0.05, **p < 0.01. Neurobehavioral recovery was assessed by three separate neurobehavioral tests including modified neurological severity score (mNSS) (**d**), rotarod performance tests (**e**), and hanging wire tests (**f**, **g**), *n* = 15–20 per group. Data are presented as mean ± SD. *p < 0.05, **p < 0.01 (vehicle vs. Bayk8644 group). ^#^*p* < 0.05, ^##^*p* < 0.01, ^###^*p* < 0.001 (*FXR* KO group vs. *FXR* KO + Bayk8644). ^&^*p* < 0.05, ^&&&^*p* < 0.001 (Bayk8644 group vs. *FXR* KO + Bayk8644 group)

To test if knockout of *FXR* exerted neuroprotective effect by inhibiting calcium influx, the calcium channel agonist Bayk8644 was injected 10 min post tMCAO (Fig. 6a). Bayk8644 treatment significantly increased infarct volume (31.8 ± 6.8 mm^3^) at 3 days after stroke and increased brain atrophy volume (8.4 ± 0.6 mm^3^) at 14 days after stroke when compared to the wild-type vehicle group. Injection of Bayk8644 in *FXR* KO mice (28.0 ± 9.4 mm^3^) illustrated larger infarct volume at 3 days after stroke and brain atrophy volume (5.3 ± 1.0 mm^3^) compared to *FXR* KO group (Fig. 6b, c). In addition, injection of Bayk8644 greatly attenuated neurobehavioral recovery at 1, 3, 7, and 14 days after tMCAO, as tested by the mNSS score (Fig. 6d), rotarod test, and (Fig. 6e) hanging wire test (Fig. 6f, g). Compared with *FXR* KO group, *FXR* KO + Bayk8644-treated mice showed worse performance in the mNSS test and rotarod test at 1, 3, and 7 days after tMCAO, and hanging wire test at 3 days (*p* < 0.001).

## Discussion

FXR, a metabolic nuclear receptor, plays a critical role in modulating systemic inflammatory responses and the maintenance of energy homeostasis [28]. Our previous study has highlighted the critical role of FXR in mediating cardiomyocytes apoptosis using a myocardial ischemia/reperfusion mouse model [8]. Here for the first time, we identified that FXR contributed to neuronal apoptosis through increasing intracellular calcium level after ischemic stroke.

In this study we first investigated the changes of FXR levels at different time points post stroke. We found that the expression of FXR was increased in the brain, specifically in neurons at 3 days after stroke, which was consistent with the acute pathologic processes of stroke when proapoptotic gene products have been implicated [29, 30]. Previous studies have shown that apoptosis in the penumbra could remain potentially salvageable, where the neurons remained metabolically active, although electrically silent [31]. Thus, reducing neuronal apoptosis may be a potential way to treat ischemic stroke. Indeed, in our present study, genetic deletion or pharmaceutical inhibition of FXR reduced neuronal apoptosis in ischemic mice and in primary neurons, while FXR agonism aggravated apoptosis in primary neurons.

It is generally acknowledged that cytokines released from inflammatory cells regulate neuronal sensitivity to stimulation and injury [32]. Inflammation process involves numerous direct effects of inflammatory cytokines on nociceptive neurons, such as changes in ion-currents, enhancement of transient receptor potential channel activity, and increases in glutamatergic effects [33]. Inflammatory cytokines, such as *TNF-α*, *IL-6*, *IL-1β*, *IL-17*, and *IL-18* can mediate the recruitment of immune cells, alteration of the blood-brain barrier (BBB), and activation of apoptotic cell death, therefore playing important roles in the pathogenesis of acute ischemic stroke [34, 35]. MPO activity is another good indicator of inflammation, and neutrophil accumulation can be quantified using the MPO activity assay. In addition, previous studies showed that FXR could modulate TLR4/MyD88-mediated inflammation and also a modulator of *NF-κB*-mediated hepatic inflammation [36, 37]. In our study, we found that *FXR* knockout reduced the expression of MPO and inflammatory cytokines levels, which may improve the microenvironment in the lesion area and thus reduce neuronal apoptosis.

Recent studies have revealed that bile acids, a ligand of FXR, promoted the accumulation of calcium within mitochondria and activate NLRP3 inflammasomes [38]. Calcium released from the endoplasmic reticulum can synchronize the mass exodus of cytochrome c from the mitochondria, a phenomenon that coordinates apoptosis [39, 40]. Thus, control of intracellular calcium signaling may be a neuroprotective strategy in both acute and chronic degenerative diseases of the nervous system [41, 42]. *FXR* knockout can block calcium influx through L-type calcium channels which attenuated mitochondrial injury and apoptosis in primary neurons. In our study, we showed that Z-GS could independently inhibit the increase in cytoplasm Ca^2+^ induced by OGD/R treatment and protect neurons. Treatment with ionomycin, which can induce greater increases in intracellular Ca^2+^concentrations, reversed the protective effect of Z-GS, supporting the hypothesis that calcium influx is a key mediator of FXR-mediated neuronal injury.

In our present study, we demonstrated that *FXR* knockout has a protective effect on cerebral ischemic injury by reducing inflammatory response and neuronal apoptosis through reducing calcium influx. The FXR inhibitor, Z-GS, reduced apoptosis in primary neurons, accompanied by reduced calcium signal. Such beneficial effects were abolished by the co-administration of the membrane-permeable calcium ionophore, ionomycin. Taken together, FXR represents a potential molecular therapeutic target for ischemic stroke.

Despite all the findings stated above, certain questions still remain unsolved such as the mechanism of activation of FXR post stroke and other effects of FXR on stroke pathophysiology in addition to protect against neuronal apoptosis, which would require further addressment in the future. One limitation of this study is that we only adopted a systemic homozygous *FXR* knockout mouse model to determine its role in neuronal apoptosis post stroke without examining the effect of knockout *FXR* in different neuron subtypes, including excitatory neurons and inhibitory neurons using conditional *FXR* knockout by Cre/loxp recombination system in experimental stroke. In addition, the effect and underlying mechanism of FXR on angiogenesis and neurogenesis during the chronic stage of stroke are also worth investigating and being incorporated into future studies.

## Conclusions

In conclusion, our results indicate that *FXR* knockout can reduce brain infarct volume, inflammation, and neuronal apoptosis and promote neurobehavioral recovery in mice after brain ischemia. FXR may be a potential therapeutic target for ischemic stroke and that may be partly related to calcium influx.

## Data Availability

The datasets used and/or analyzed during the current study are available from the corresponding author upon reasonable request.

## Abbreviations

BAX: BCL2-associated X protein
BCA: Bicinchoninic acid
BCL-2: B cell lymphoma-2
BDNF: Brain-derived neurotrophic factor
BSA: Bovine serum albumin
CAMKII: Ca^2+^/calmodulin (CaM)-dependent protein kinase II
CCK8: Cell counting kit-8
CD31: Cluster of differentiation 31
CNS: Central nervous system
CYP7A1: Cytochrome P450 7A1
DAPI: 4′,6-Diamidino-2- phenylindole dihydrochloride
DMEM: Dulbecco’s modified eagle medium
DMSO: Dimethyl sulfoxide
ECA: External carotid artery
FXR: Farnesoid X receptor
FBS: Fetal bovine serum
GAD65: Glutamic acid decarboxylase 65
GAPDH: Glyceraldehyde phosphate dehydrogenase
GAT1: GABA transporter 1
GFAP: Glial fibrillary acidic protein
Iba-1: Ionized calcium binding adaptor molecule-1
ICA: Internal carotid artery
IL-1β: Interleukin-1β
IL-6: Interleukin-6
IL-17: Interleukin-17
IL-18: Interleukin-18
KO: Knockout
MCA: Middle cerebral artery
MPO: Myeloperoxidase
mNSS: Modified neurological severity score
mRNA: Message ribonucleic acid
NR1H4: Nuclear receptor subfamily 1 group H member 4
OGD: Oxygen-glucose deprivation
PBS: Phosphate-buffered saline
PFA: Paraformaldehyde
RT-PCR: Reverse transcription-polymerase chain reaction
SDS-PAGE: Sodium dodecyl sulfate-polyacrylamide gel electrophoresis
TBST: Tris-buffered saline tween
tMCAO: Transient middle cerebral artery occlusion
tPA: Tissue plasminogen activator
TUNEL: Terminal dexynucleotidyl transferase mediated dUTP nick end labeling
Z-GS: Z-Guggulsterone
